# Power and false-positive rates for the restricted partition method (RPM) in a large candidate gene data set

**DOI:** 10.1186/1753-6561-3-s7-s74

**Published:** 2009-12-15

**Authors:** Robert Culverhouse, Wu Jin, Carol H Jin, Anthony L Hinrichs, Brian K Suarez

**Affiliations:** 1 Department of Medicine, Washington University School of Medicine, 660 South Euclid Avenue, St. Louis, Missouri 63110, USA; 2 Department of Psychiatry, Washington University School of Medicine, 660 South Euclid Avenue, St. Louis, Missouri 63110, USA; 3 Department of Genetics, Washington University School of Medicine, 660 South Euclid Avenue, St. Louis, Missouri 63110, USA

## Abstract

Many phenotypes of public health importance (e.g., diabetes, coronary artery disease, major depression, obesity, and addictions to alcohol and nicotine) involve complex pathways of action. Interactions between genetic variants or between genetic variants and environmental factors likely play important roles in the functioning of these pathways. Unfortunately, complex interacting systems are likely to have important interacting factors that may not readily reveal themselves to univariate analyses. Instead, detecting the role of some of these factors may require analyses that are sensitive to interaction effects.

In this study, we evaluate the sensitivity and specificity of the restricted partition method (RPM) to detect signals related to coronary artery disease in the Genetic Analysis Workshop 16 Problem 3 data using the 50,000 k candidate gene single-nucleotide polymorphism set. Power and false-positive rates were evaluated using the first 100 replicate datasets. This included an exploration of the utility of using of all genotyped family members compared with selecting one member per family.

## Background

Coronary artery calcification (CAC) is a quantitative trait that is a consistently found in areas of coronary artery narrowing. In the Genetic Analysis Workshop (GAW) 16 simulation, CAC was used as a surrogate for plaque build-up as a risk factor for a heart attack. Coronary event (CE), e.g., myocardial infarction, was a binary trait included in the data. Because the Framingham Heart Study sample was not ascertained as a case-control study and CEs were relatively rare in the data, we expected the data would have less power to detect factors contributing CE independently from CAC than it would to detect factors contributing directly to CAC. We note that lipids (high-density lipoprotein, low-density lipoprotein, and triglycerides as well as the composite score, total cholesterol) are well known factors associated with coronary artery disease.

## Methods

The restricted partition method (RPM) uses multiple comparisons to evaluate whether the phenotypes associated with each (multilocus) genotype in a particular model (e.g., a two-SNP model consisting of nine genotypes) come from the same distribution. If the answer is "no", the method proposes a partition of the genotypes. The test statistic is the proportion of the trait variation explained by the partition. The statistic's significance is determined by permutation testing. Because the RPM is appropriate for either quantitative or binary phenotypes [1,2], we chose to analyze both the quantitative trait CAC and the binary trait CE. In order to improve our ability to detect factors contributing directly to CAC, our phenotype for analysis was the residual after regression involving age, sex, total cholesterol, and high-density lipoproteins. The current version of the RPM does not allow for adjustments for covariates when a binary trait is being analyzed. Thus, our analysis of the binary trait CE did not include adjustments for these factors.

The RPM was developed as a method for analyzing datasets consisting of unrelated subjects. Since the Framingham Heart Study data is family-based, one goal of this study was to compare the utility of naively including all subjects in an RPM analysis to that of analyzing a subset of the data consisting of only one subject per family. A second question addressed by these analyses is whether true signals could be distinguished from noise in pairwise analyses of a large set of candidate SNPs.

We knew the true model before starting our analyses.

### CAC model

The true model for CAC involved the lipids (which were modeled using genetic variants from the 500,000 genome-wide association study [GWAS] SNP set), age, and five SNPs from the 50,000 candidate gene SNP set that directly affected CAC. One of these SNPs, Het, acted independently of the others and displayed over-dominance (heterosis). SNPs PE1 and PE2 interacted with each other to affect CAC, but theoretically would not display any effects when analyzed separately. SNPs ME1 and ME2 acted primarily through an interaction, but ME2 displayed a measurable effect when analyzed alone, while ME1 did not. Perhaps unrealistically, the dependence of CAC on lipids was modeled in such a way that exercise and lipid-lowering medication could decrease CAC.

### CE model

The primary risk factor for CE was CAC. Thinking of CAC as a surrogate for plaque build-up, the constricting effect of smoking on arteries can be viewed as if smoking effectively increased CAC. This was how the effect of smoking was incorporated in the generating model. The model included two additional polymorphisms that modified risk: SLoc modified of risk only in smokers; EventLoc had a small direct effect on risk for all subjects.

For a complete description of the simulation model including a diagram illustrating the relationships between predictors and phenotypes, see Kraja et al. [3].

### Data

Because CAC only develops in middle age, but is affected by lipid-lowering medication, we restricted our analyses on data from the second examination of each subject in the hope of maximizing the variance in CAC due to genetic effects. Because of the computational complexity of analyzing all pairwise interactions, we used only the first 100 data replicates and the 50,000 candidate gene SNP set for our analysis.

Analyses were first run on the full dataset of 6479 subjects, ignoring family structure. Next, analyses were run on a subset consisting of just one member of each family, the oldest subject (*N *= 1130). We will refer to these two datasets as the "full data" and the "unrelateds data," respectively. The goal of this dual analysis approach was to evaluate the benefits of increased sample size versus the potential increase of false positives when including related individuals in an analysis intended for unrelated (or very distantly related) individuals. The two datasets had similar distributions of the sexes (45.7% male in the full data, 46.6% male in the unrelateds) and smoking (21.2% of women, 24.9% of men smoked in the full data, 21.3% of women and 25.0% of men smoked among the unrelateds). Because of the ascertainment strategy, the average age in the "Unrelateds" dataset was greater than that in the full dataset (63.1 vs. 52.6) and the CE rate was higher (11.8% vs. 7.7%). If this difference has an effect, it would be expected to increase to power of the "Unrelateds" data to detect factors related to CE.

For each of the first 100 data replicates, the true contributing factors to the two traits were evaluated using the RPM, using univariate or two-way analyses as appropriate. The proportion of trait variation explained by the model and a permutation *p*-value based on 10,000,000 permutations were recorded for each.

To estimate the false-positive rates, we began by cleaning the data as we would for any analysis: removing all monomorphic SNPs (defined by fewer than 0.1% heterozygous subjects) and SNPs with a high proportion of missing genotypes (missing in more than 700 subjects in the full data, missing in more than 100 in the unrelateds). Because signals associated with SNPs in linkage disequilibrium (LD) with the true causative factors for CAC and CE should not be considered "false" positives, we removed the few SNPs displaying LD with any causative SNP (*r*^2 ^> 0.1) from this portion of the analysis. This left 42,461 SNPs for the analysis of false positives in the "All Subjects" dataset, and 41,101 SNPs in the "Unrelateds" dataset. Univariate RPM analyses were run on all these SNPs for each of the two traits in each of the 100 replicate datasets. *p*-Values were estimated from 1,000,000 permutations per SNP. To save computation time, the false-positive distribution for pairwise analyses was not estimated by exhaustively evaluating all of the nearly 1 billion pairs of null SNPs in each replicate. Instead, the distribution was estimated from a random sampling of 1,000,000 null pairs chosen independently from each of the 100 replicates (for a total 100,000,000 pairs).

## Results

Results from the RPM analysis of both datasets using the second visit can be found in Tables 1 and 2. The RPM provides two measures of "signal": the model *R*^2 ^and a permutation-based *p*-value. Typically, these would be used jointly to enhance computational efficiency. The tables contain results from using different thresholds on the two measures as tests of significance.

**Table 1:** Power and false-positive rates: all subjects (*N *= 6479)

CAC	*p*-value Threshold					*R*^2 ^Threshold					*R*^2 ^Mean^a^
			
	10^-3^	10^-4^	10^-5^	10^-6^	10^-7^	0.005	0.01	0.02	0.03	0.04	
Univariate											
**Power^b^**											
Het	100	100	100	100	100	100	100	100	29	0	0.0279
ME1	0	0	0	0	0	0	0	0	0	0	0.0001
ME2	85	69	50	32	12	12	0	0	0	0	0.0034
PE1	0	0	0	0	0	0	0	0	0	0	0.0001
PE2	0	0	0	0	0	0	0	0	0	0	0.0001
**Avg(#FP)^c^**	89.8	6.9	3.2	0.9	0.1	3.3	1	1	0.3	0	
**E(#FP)^d^**	42.5	4.2	0.4	0	0						
											
Two-way											
**Power**											
ME1*ME2	100	100	100	100	100	100	100	100	100	100	0.0539
PE1*PE2	100	100	100	100	100	100	100	100	100	100	0.1095
**Avg(#FP)**	1.6 M	202 K	28 K	3484	631	68 K	175	<10	<10	<10	
**E(#FP)**	901 K	90 K	9014	901	90						
											
Univariate											
**Coronary Event**											
**Power**											
Het	68	42	28	19	11	13	0	0	0	0	0.0026
ME1	0	0	0	0	0	0	0	0	0	0	0.0001
ME2	3	0	0	0	0	0	0	0	0	0	0.0003
PE1	0	0	0	0	0	0	0	0	0	0	0.0001
PE2	0	0	0	0	0	0	0	0	0	0	0.0001
Smoke	98	96	87	71	56	47	1	0	0	0	0.0049
SLoc	0	0	0	0	0	0	0	0	0	0	0.0002
Event Loc	11	2	0	0	0	0	0	0	0	0	0.0008
**Avg(#FP)**	80.4	5.1	4.6	3.6	2.1	3.2	2	2	1.3	0	
**E(#FP)**	42.5	4.2	0.4	0	0						
											
Two-way											
**Power**											
ME1*ME2	100	99	98	86	82	97	29	0	0	0	0.0089
PE1*PE2	100	100	100	100	100	100	100	40	0	0	0.0193
Smoke*SLoc	99	95	87	63	47	77	5	0	0	0	0.0069
**Avg(#FP)**	1.5 M	173 K	21 K	925	90	96 K	174	<10	<10	<10	
**E(#FP)**	901 K	90 K	9014	901	90						

^a^Mean RPM *R*^2 ^for the factors in the model estimated by the RPM in 100 data replicates

^b^Power, number of replicates for which the factor passed the threshold

^c^Avg(#FP), average number of observed "false positives" in the univariate analyses per replicate. For two-way analyses, estimated from 10^8 ^sampled pairs for the analyses of *R*^2 ^and 10^7 ^sampled pairs for the analyses of *p*-values.

^d^E(#FP), expected number of false positives in the analysis of one replicate assuming the tests were independent.

**Table 2:** Power and false positive rates - unrelated subjects (*N *= 1130)

CAC	*p*-value Threshold					*R*^2 ^Threshold					*R*^2 ^Mean^a^
			
	10^-3^	10^-4^	10^-5^	10^-6^	10^-7^	0.005	0.01	0.02	0.03	0.04	
Univariate											
**Power^b^**											
Het	100	100	100	100	97	100	100	100	95	68	0.0447
ME1	0	0	0	0	0	9	0	0	0	0	0.0011
ME2	2	0	0	0	0	22	5	0	0	0	0.0027
PE1	0	0	0	0	0	6	0	0	0	0	0.0007
PE2	0	0	0	0	0	1	0	0	0	0	0.0002
**Avg(#FP)^c^**	49.0	7.5	1.3	0.1	0.03	757.4	89.2	2.3	1.0	0.6	
**E(#FP)**	41.1	4.1	0.4	0.0	0.0						
											
Two-way											
**Power**											
ME1*ME2	100	100	100	100	100	100	100	100	100	100	0.0806
PE1*PE2	100	100	100	100	100	100	100	100	100	100	0.1187
**Avg(#FP)**	1.8 M	400 K	3634	1183	422	120 M	24 M	513 K	13.3 K	550	
**E(#FP)**	845 K	84 K	8446	845	84.5						
											
Univariate											
**Coronary Event**											
**Power**											
Het	4	2	0	0	0	44	15	2	0	0	0.0041
ME1	0	0	0	0	0	4	0	0	0	0	0.0007
ME2	1	0	0	0	0	4	1	0	0	0	0.0010
PE1	0	0	0	0	0	1	0	0	0	0	0.0003
PE2	0	0	0	0	0	4	0	0	0	0	0.0006
Smoke	24	12	4	2	1	63	25	4	1	0	0.0068
SLoc	0	0	0	0	0	8	1	0	0	0	0.0012
Event Loc	0	0	0	0	0	18	1	0	0	0	0.0017
**Avg(#FP)**	36.7	4.5	0.8	0.1	0.01	631.1	69.0	1.8	0.1	0.0	
**E(#FP)**	41.1	4.1	0.4	0.0	0.0						
											
Two-way											
**Power**											
ME1*ME2	43	16	7	1	0	98	88	43	6	0	0.0183
PE1*PE2	94	75	45	25	8	100	100	92	51	9	0.0301
Smoke*SLoc	25	7	4	1	0	81	54	12	3	0	0.0114
**Avg(#FP)**	746 K	83 K	9740	342	84	100 M	18 M	402 K	15.6 K	967	
**E(#FP)**	845 K	84 K	8446	845	84.5						

^a^Mean RPM *R*^2 ^for the factors in the model estimated by the RPM in 100 data replicates

^b^Power, number of replicates for which the factor passed the threshold

^c^Avg(#FP), average number of observed "false positives" in the univariate analyses per replicate. For two-way analyses, estimated from 10^8 ^sampled pairs for the analyses of *R*^2 ^and 10^7 ^sampled pairs for the analyses of *p*-values.

^d^E(#FP), expected number of false positives in the analysis of one replicate assuming the tests were independent.

Table 1 contains results from the analyses including all the subjects. In the analysis of the CAC phenotype, the univariate signal from the over-dominant locus Het was easily detected in the univariate analysis. Also, although it had a smaller effect size, the epistatic locus ME2, which was designed to display a small main effect, was detectable as well. As expected, univariate analysis had no power to detect the loci designed to have purely epistatic effects (ME1, PE1, and PE2).

When analyzing the data containing all the subjects, the effect sizes due to the epistatic interactions were well above the level of background noise. For instance, no randomly sampled pair explained more than 1% of the trait variance. In contrast, the two interacting pairs could, on average, account for approximately 5% and 11% of the trait variance, respectively.

Because only 100 million pairs of SNPs were sampled from the approximately 901 million possible pairs of SNPs in this data, the average number of false positives for the two-way analyses were approximated by multiplying the observed number of false positives by 9.01. If no false positives were observed for a given threshold, our best estimate is that we could expect fewer than 10 false positives if a full two-way analysis of all the SNPs were to be performed.

The CAC loci produce a weaker signal in the CE phenotype, but the interactions are still detectable, as is smoking.

Table 2 presents results parallel to those of Table 1, but is based on analysis of the smaller dataset consisting of just one person per pedigree. As expected, the power is decreased for most thresholds. In addition, the test statistics for both the true models and models based on random noise have increased variance. In this case, the level of background noise is comparable to the true signals.

## Conclusion

The first point suggested by these data relates to including genotyped relatives in an analysis designed for unrelated individuals. It is often argued that the conservative approach is to select one subject per family, because including relatives in such an analysis can give rise to false-positive signals. Others argue that as long as the family size does not vary dramatically in the sample (leading to a few families driving the results), the increased power gained by including related subjects can be worth the loss of an accurate estimate of effect-size.

In the GAW16 simulated data, based on the true Framingham Heart Study genotypes and family structure, analysis using the RPM lends support to the latter argument. In this case, including related individuals in the analysis caused only a moderate increase in false positives, but greatly increased the power to find true signals. This proved to be particularly useful in these data because the signals associated with the phenotypes were particularly modest: with 1130 unrelated individuals, many of the signals did not exceed the level of background noise; with 6479 related individuals, they did.

As we continue to try to determine factors contributing to common, complex phenotypes we will likely continue to find that we are looking for factors of modest effect size. Our results suggest that for a sufficiently large gain in sample size, it may be worth violating the assumption of unrelatedness.

A second point relates to search strategies for detecting interacting trait predictors. All such strategies carry considerable computational and statistical costs. Given the design of the generating model [3], one can see *a priori *that the cheapest approach, namely testing for interactions among the top univariate signals, would almost certainly have failed to detect either of the interactions in the models for CAC and CEs. Similarly, the intermediate method of testing a top univariate result against all other predictors might have found one of the interactions, depending on how low into the univariate list one was willing to go. However, it is extremely unlikely that both of the interactions could have been found without some sort of exhaustive search through all pairs.

Finally, we note the effect of missing data. In these analyses we included SNPs that had approximately a 90% call rate. It is now common to require call rates of at least 98% for a SNP to be included in analysis. Although this has not proved to be a problem for recent datasets we have worked with, in the Framingham Heart Study Candidate Gene data it would have eliminated nearly 25% of the SNPs. However, we also found that lowering the call rate threshold caused a rapid increase in false-positive calls. We are not certain if this is due simply to the instability of the models produced from small samples or if the data from the low call rate SNPs are truly unreliable.

## List of abbreviations used

CAC: Coronary artery calcification; CE: Coronary event; GAW: Genetic Analysis Workshop; GWAS: Genome-wide association study; LD: Linkage disequilibrium; RPM: Restricted partition method; SNP: Single-nucleotide polymorphism

## Competing interests

The authors declare that they have no competing interests.

## Authors' contributions

RC conceived the study and drafted the manuscript. ALH and BKS assisted in the design and methodology. WJ and CHJ assisted in statistics and data management. All authors read and approved the final manuscript.
